# The detrimental effects of microplastic exposure on kidney function

**DOI:** 10.3389/fmed.2025.1620733

**Published:** 2025-09-23

**Authors:** Na Jiang, Xiya Zheng, Nan Zhang, Yingjie Cao

**Affiliations:** Department of Nephrology, The Afffliated Hospital of Nantong University, Medical school of Nantong University, Nantong, China

**Keywords:** microplastics, apoptosis, autophagy, inflammatory-response pathways, renal fibrosis

## Abstract

Microplastics (MPs) are plastic particles with a diameter of less than 5 millimeters, primarily originating from the degradation of plastic products (11). In recent years, increasing attention has also been given to the impact of MPs on the health. Important questions have surfaced, including whether MPs can be cleared by the kidneys, whether reduced kidney function affects their clearance, and whether MP accumulation contributes to the progression of kidney diseases. This review explores the effects of MPs on the kidneys and focuses on their accumulation, toxic effects, and potential molecular mechanisms.

## Introduction

MPs are organic polymers smaller than 5 millimeters in size with varying shapes, while Nps are polymers smaller than 1 micrometer in size (1). Due to the long degradation time of plastics and the high volume of daily production, MPs and Nps are ubiquitous in modern life (2).

In 1909, Belgian chemist Leo Hendrik Baekeland invented plastic, which has since been in use for over a century (3). Plastics are now widely integrated in our daily lives. In medicine, plastics are extensively used in items such as test tubes, surgical drapes, catheters, probes, and intravenous or arterial access devices (4). However, the widespread presence of plastics has led to global pollution and plastic-related waste. For instance, it is estimated that over 250,000 tons of plastic-related waste have accumulated in the ocean. Through degradation or mechanical processes, plastics break down into small particles known as MPs or nanoplastics (Nps) (1).

In fact, as early as the 1970s, Ed Carpenter had found tiny plastic particles in the ocean (5). In 2004, Thompson first proposed the term “microplastic” and clearly defined it as plastic fragments smaller than 5 mm (6).

In 2018, microplastics were found in human feces for the first time (7). Subsequently, microplastics were detected in the placenta, lungs, blood and even breast milk. This confirms that humans are inevitably exposed to a microplastic environment, and has raised concerns about the impact of microplastics on human health (8).

In recent years, the environmental impact and health risks of MPs have become a focus of research. Studies have shown that MPs can enter the food chain, affect the growth and development of organisms, and even alter the structure and function of ecosystems (9). Of particular concern is the ability of MPs to enter the circulatory system and penetrate cell membranes (10), causing damage to multiple organs, including the kidneys (11). For example, exposure to polystyrene microplastics (PS-MPs) has been linked to pulmonary toxicity (12), cardiotoxicity (13), reproductive toxicity (14), neurotoxicity (15), hepatotoxicity (16), and intestinal toxicity (17). As a crucial excretory and metabolic organ, kidney health is closely tied to overall well-being.

The detection of nanoplastics requires expensive and complex instruments such as electron microscope, Raman spectroscopy and there is a lack of standardized detection methods. Microplastics are more extensive research. This review explores the impact of MPs on kidney health, examines their potential toxic mechanisms, and discusses the advances and challenges of current research. We conduct a comprehensive analysis covering MP sources and types, their bioaccumulation, and associated toxic effects. The goal is to provide a scientific basis for understanding the environmental risks of MPs and to inform in the development of effective prevention and control measures.

## Common methods for detecting MPs

Currently, there is no single analytical method that can qualitatively or quantitatively detect all types of MPs. Different types of microscopes, such as stereomicroscopes, electron microscopes, and fluorescence microscopes, are used as physical detection tools to distinguish MPs from other substances.

Spectroscopic analysis techniques, such as Fourier-transform infrared spectroscopy (FTIR) and Raman spectroscopy, can differentiate plastics from additives based on their composition. FTIR identifies the polymer types by analyzing their infrared absorption spectra. This method requires additional sample-preparation steps, including air drying and screening. Raman spectroscopy, on the other hand, provides spectral information for identifying MP polymers. This non-destructive technique has the advantage of directly analyzing MPs in complex samples, making it a valuable method for in-situ analysis (18, 19).

Mass spectrometry has emerged as a valuable technique for detecting and characterizing the molecular composition of MPs. In this method, MPs are fragmented and ionized to measure the mass-to-charge ratio of the generated ions, which are produced using an oxidation furnace under oxygen flow. Gas chromatography is another technique that involves several sample preparation steps, including sampling, digestion, filtration, pressurized liquid extraction, and gas chromatographic separation based on compound volatility. This method is well-suited for analyzing volatile organic compounds associated with MPs, as well as identifying and quantifying organic additives and degradation products. When gas chromatography is combined with mass spectrometry, researchers can effectively separate and characterize MPs and related pollutants (19).

**Figure 1:**
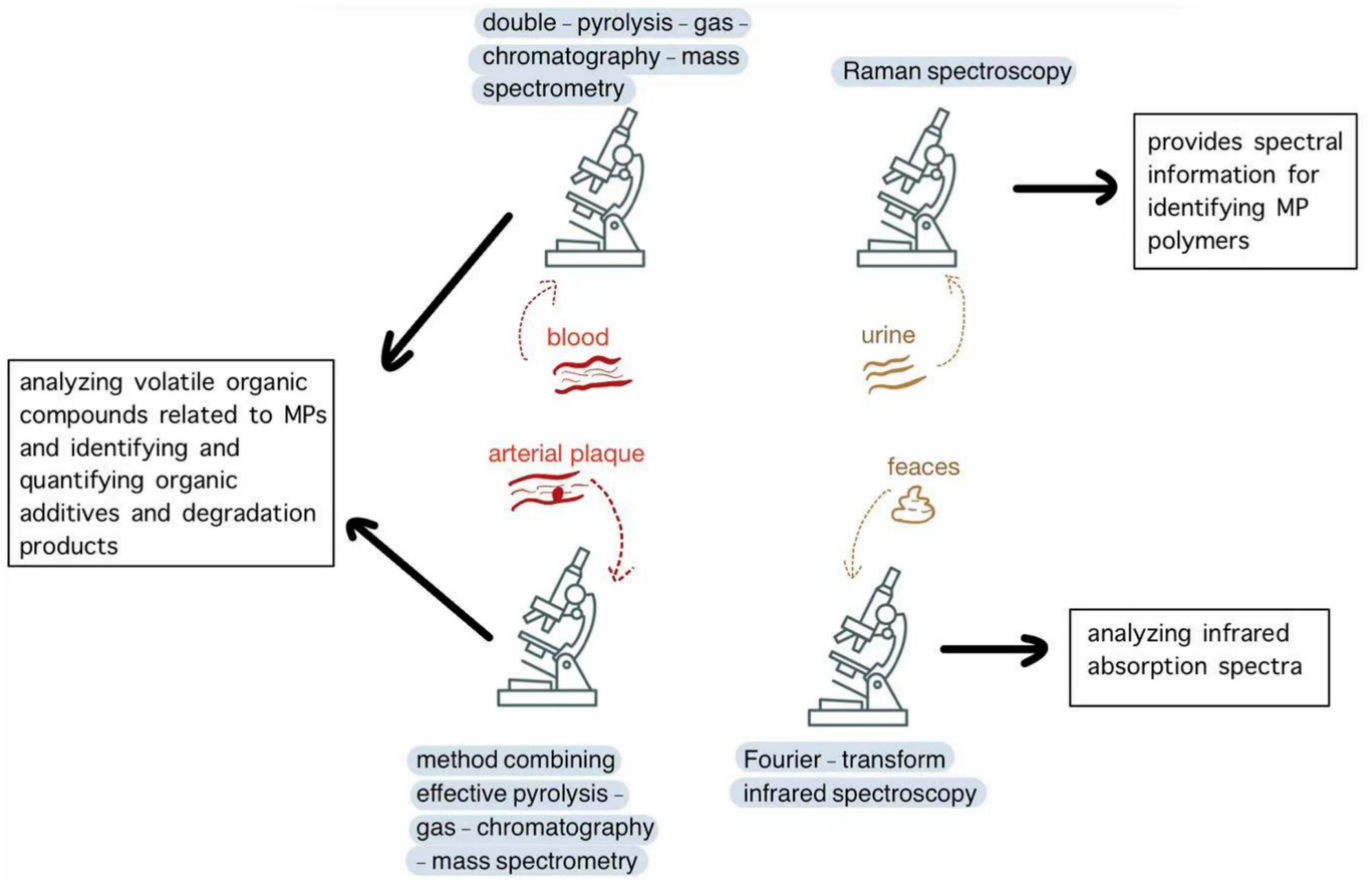
Common methods for detecting MPs.

Different sample types require different detection methods. In blood samples, MPs are detected using double-pyrolysis gas chromatography–mass spectrometry (20); in urine, Raman spectroscopy is used (21); and in feces, Fourier-transform infrared spectroscopy is applied (22). For tissue samples, such as the liver, kidneys, and spleen, detection involves digestion, staining with Nile red, and analysis by fluorescence microscopy and Raman spectroscopy (23). In patients with carotid artery plaques, MPs and nanoparticles are analyzed using a combination of pyrolysis gas chromatography–mass spectrometry, stable isotope analysis, and electron microscopy (24) (Figure 1).

## Metabolism and effects of MPs in the body

Several studies have demonstrated the presence of MPs in the kidneys of animal models, and urinary excretion has been identified as a potential route of their elimination. Recent research has also confirmed the presence of MPs in human urine and kidney tissue (21). One study in male mice investigated the single-dose administration of fluorescent polystyrene beads (100 nm and 3 μm in diameter) via tail-vein injection, gastric perfusion, or lung perfusion. The researchers proposed a mechanism to explain how, due to their small molecular size, MPs in the blood can freely pass through the filtration barrier of the glomerulus, enter the peritubular capillaries through the efferent arteriole, be taken up by the epithelial cells of the proximal tubule through endocytosis or pinocytosis, and then be secreted into the lumen and excreted in urine (25).

*In vitro* and *in vivo* studies have shown that MPs cause dysregulation of inflammatory molecules, such as interleukins, tumor necrosis factors, chemokines, transcription and growth factors, as well as cause oxidative stress. In addition, MPs and nanoplastics can carry or encapsulate heavy metals and other organic compounds into the human body, functioning as a Trojan horse, thereby increasing the risk of cancer (26). Phthalates, such as dibutyl phthalate (DBP), and other additives used in plastic manufacturing can contribute to the toxicity. Huo et al. found that DBP induces liver damage through multiple pathways (27). In general, the toxicity of MPs and nanoplastics is also related to the additives used in its production process. Another study revealed that a significant amount of inorganic pollutant leached from MPs are additive-derived (28).

**Figure 2:**
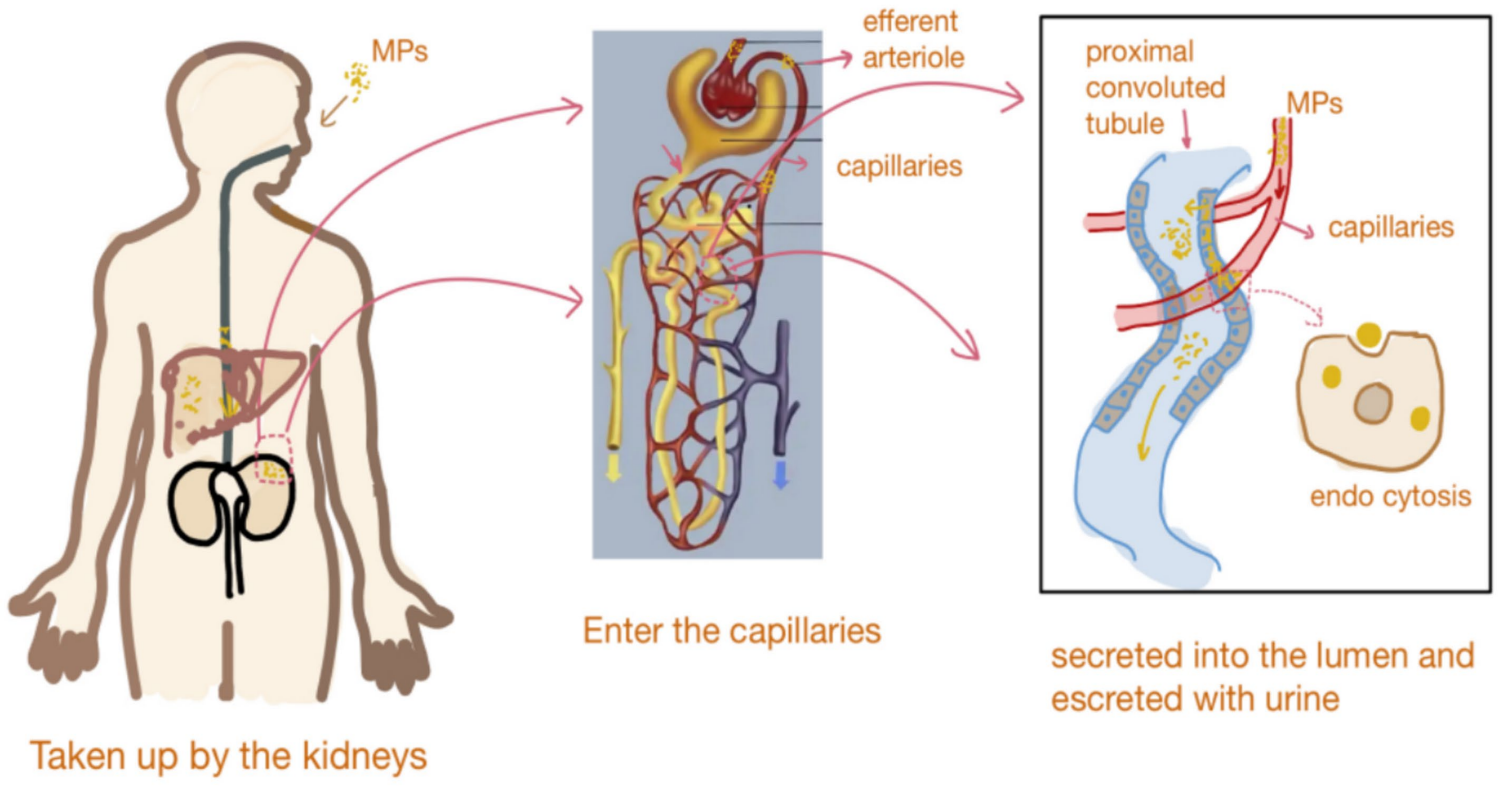
Metabolism and effects of MPs in the body.

Studies have confirmed that MPs can accumulate in multiple organs. In 2022, scientists detected MPs in human blood for the first time and observed their accumulation in blood vessels, posing potential risks to the cardiovascular system (9). Research has also shown that MPs can disrupt the endocrine system, affecting organs such as the thyroid, testes, ovaries, pituitary gland, and adrenal glands (29). Therefore, MPs may contribute to the development of chronic diseases, including obesity, diabetes, and cancer. In addition, studies in mouse models suggest that MPs can impair the self-renewal capacity of hematopoietic stem cells (HSC). Jiang et al. established a murine model for long-term MP ingestion and found that MPs caused severe damage to the hematopoietic system. Fecal microbiota transplantation (FMT) from mice orally exposed to MPs significantly impaired the self-renewal and reconstitution capacity of hematopoietic stem cells (HSCs). Mechanistically, MPs did not directly kill HSCs but disrupted intestinal integrity and barrier function, ultimately increasing the abundance of Rikenellaceae and hypoxanthine in the gut while inactivating the HPRT-Wnt signaling pathway in bone marrow HSCs (30) (Figure 2).

## Effects of MP exposure on kidney structural changes

The kidney is a vital organ responsible for maintaining fluid and electrolyte balance through various processes (31). Podocytes in the glomerulus are essential for maintaining the integrity and function of the glomerular filtration barrier (32). MPs can damage podocytes, disrupting their structure and function. For example, studies using kidney organoids have shown that MPs induce fusion of podocyte processes, thereby damaging the glomerular filtration barrier. This damage allows macromolecules, such as proteins, to leak into the urine, leading to proteinuria and impaired kidney function (33–35).

MP exposure may also result in mitochondrial cristae rupture or vacuolization, nuclear membrane wrinkling, and chromatin aggregation (36). Mitochondrial damage disrupts cellular energy production, thereby affecting critical physiological activities such as biosynthesis, transport, and signal transduction. MPs have also been shown to cause thickening of the glomerular basement membrane and deposition of fibrous tissue in the mesangial area. In kidney tubules, MPs induce vacuolar and granular degeneration, atrophy, and necrosis, affecting reabsorption and secretion. Changes in cell morphology, as well as increased apoptosis and autophagy, may occur in epithelial cells of kidney tubules, leading to selective reabsorption and excretion of substances and disrupting water-salt balance and metabolic waste excretion (37). Chronic MP exposure may lead to tubular atrophy, narrowed tubular lumens, reduced epithelial cell numbers, elevated numbers of autophagosomes and lysosomes, and increased lipid droplet accumulation, interstitial fibrosis, and deposition of collagen fibers in the kidney interstitium (38, 39).

## Effects of MPs on the glomerulus

In kidney organoids, podocytes may be affected by MP exposure, compromising their structure and affecting the function of the glomerular filtration barrier. Zhou et al. treated kidney organoids with 1-μm PS-MPs for 48 h. Transmission electron microscopy revealed vacuoles in the cytoplasm, condensation of the cytoplasm, nuclear fragmentation, and increased number of autophagolysosomes. They also found that reactive oxygen species (ROS) may directly or indirectly regulate the expression of the WT-1 gene, resulting in its downregulation (40). Under physiological conditions, podocytes form a sieve-like structure through complex intercellular connections to selectively filter plasma components. PS-MPs disrupt these connections, affecting the structural stability of the glomerulus. Wang et al. confirmed that PS-MPa impair glomerular filtration, causing macromolecules, such as proteins, to leak into the urine and triggering proteinuria (35).

Tan et al. demonstrated that PS-NPs induce an inflammatory response in the kidneys of mice. Microplastics activate immune cells in the glomerulus, such as macrophages and neutrophils, causing them to release inflammatory factors such as tumor necrosis factor (TNF) and interleukin (IL). These cytokines can stimulate glomerular endothelial and mesangial cells, triggering cell proliferation and phenotypic changes, and then leading to increased CD34 expression (41), a marker of cell proliferation and angiogenesis. Inflammatory factors upregulate the expression of CD34 by activating signaling pathways, notably nuclear factor NF-κB and mitogen-activated protein kinase (MAPK) signaling pathways, either directly or indirectly. For example, NF-κB translocates into the nucleus and binds to specific sequences in the promoter region of the CD34 gene to promote its transcription. In the MP-induced inflammatory environment, these signaling pathways in glomerular cells are activated, resulting in increased CD34 expression, which may be related to changes in cell proliferation and angiogenesis.

## Effects of MPs on kidney tubules

### Uptake and accumulation of MPs by epithelial cells of kidney tubules

A previous study showed that PS-MPs accumulate both *in vitro* (in HK-2 cells) and *in vivo* (in mice). The uptake of PS-MPs by HK-2 cells at different concentrations associates with higher mitochondrial ROS levels and increased expression of the ER stress-related protein Bad (35). Recently, Wang et al. (42) found that HK-2 cells showed a time-dependent uptake pattern when co-incubated with different concentrations (0.4 mg/mL and 0.8 mg/mL) of PS-MPs. Using nanoparticle-tracking analysis, they observed that the amount of PS-MPs uptaken by cells gradually increased over 24 h. At a concentration of 0.8 mg/mL, the number of extracellular vesicles (EVs) at 24 h was approximately 4.3 times that of the control group, indicating continued accumulation of PS-MPs. At the same time point, uptake was significantly higher in the 0.8 mg/mL treatment group compared to the 0.4 mg/mL treatment group. The above results indicate that the accumulation of MPs in the kidney is time-dose dependent.

### Effects of MPs on the viability of epithelial cells in kidney tubules

Goodman et al. exposed human embryonic kidney cells to PS-MPs and found that they significantly reduced the expression of superoxide dismutase (SOD) and catalase (CAT) in kidney cells, thereby increasing ROS levels and reducing cell viability (43). Cells exposed to 1-μm PS-MPs at a concentration of up to 100 μg/mL maintained a viability of at least 94%, as determined by trypan blue exclusion. These results indicate that, although PS-MPs induced oxidative stress, they retained a certain degree of viability, possibly due to intrinsic stress or defense mechanisms that can mitigate PS-MP-induced cellular damage.

Wang et al. (42) exposed cells to different concentrations (5 μg/mL, 50 μg/mL, and 100 μg/mL) of PS-MPs and measured ROS levels at multiple time points (0, 2, 4, 6, 12, and 24 h). They found that ROS levels increased with both PS-MP concentration and exposure duration. At 50 μg/mL, a significant increase in ROS levels was detected 2 h after treatment, with levels remaining elevated or continuing to increase over time. Multiple studies have reported dose-and time-dependent increases in mitochondrial ROS levels in HK-2 cells after PS-MP exposure.

## Mechanisms of the toxic effects of microplastics on the kidneys

### Oxidative stress induced by MPs on kidney cells

Research shows that MPs increase ROS levels in kidney cells, thereby activating inflammatory signaling pathways (26). Ahmed et al. evaluated the toxic effects of PS-NPs on the kidneys of adult male albino rats and found that PS-NPs significantly reduced the expression of (GSH) and glutathione peroxidase (GPX), leading to excessive ROS production and oxidative stress in the kidneys (44). In addition, Shen et al. reported that long-term exposure to environmentally relevant concentrations of PS-MPs significantly upregulated the expression of mitochondrial-related genes, particularly those involved in thermogenesis and oxidative phosphorylation, resulting in kidney damage (45). Therefore, MPs can exacerbate nephrotoxicity by inducing mitochondrial dysfunction and disrupting oxidative balance in the kidneys.

### Activation of inflammatory-response pathways induced by MPs

Research indicates that MPs can activate inflammatory signaling pathways, such as the NF-κB pathway, leading to an increase in the expression of inflammatory factors (46). Wang et al. found that ROS activates the ER-stress marker GRP78, increases CHOP expression through the IRE1/XBP1s and ATF6 pathways, promotes JNK signaling, and further activates NF-κB, exacerbating the inflammatory response (34). Studies also show that MPs cause histological damage to the kidneys by affecting serum urea nitrogen and creatinine levels, and promoting the release of inflammatory mediators such as IL-1β, IL-6, and TNF-*α*. In addition, the activation of the NLRP3 inflammasome recruits ASC and caspase-1, further activates caspase-1, and promotes the secretion of pro-inflammatory factors, exacerbating kidney inflammation and fibrosis (47).

### Activation of cell apoptosis by MPs

Li et al. reported that MPs can induce oxidative stress *in vivo*. The resulting ROS can cause oxidative damage to the endoplasmic reticulum (ER) and its associated membrane proteins, further exacerbating ER damage and leading to ER stress (36). Wang et al. studied and showed that PSMPs activate the GRP78–IRE1–XBP1s and ATF6 pathways, both of which underlie ER stress, by promoting the accumulation of ROS and resulting in increased CHOP expression. This activation induces apoptosis by activating caspase-12, caspase-9, and caspase-3. Concurrently, the upregulation of the pro-apoptotic protein Bax and the downregulation of the anti-apoptotic protein Bcl-2 further promote apoptosis (34). In addition, Li et al. found that PS-NPs and lipopolysaccharides, either individually or in combination, induce ER stress through oxidative stress. This activates the IRE1/XBP1 pathway, leads to ER stress, and promotes the expression of caspase-3 and caspase-12, ultimately promoting apoptosis (36). Recently, Chen et al. exposed HK-2 cells to PS-NPs and found that NR4A1 translocated from the nucleus to the mitochondria, which disrupted the mitochondrial membrane potential, released cytochrome C, activated Caspase-3, and ultimately induced apoptosis (48). In summary, MPs, either alone or in combination with other agents, can induce apoptosis through multiple pathways.

### Activation of renal fibrosis by MPs

**Figure 3:**
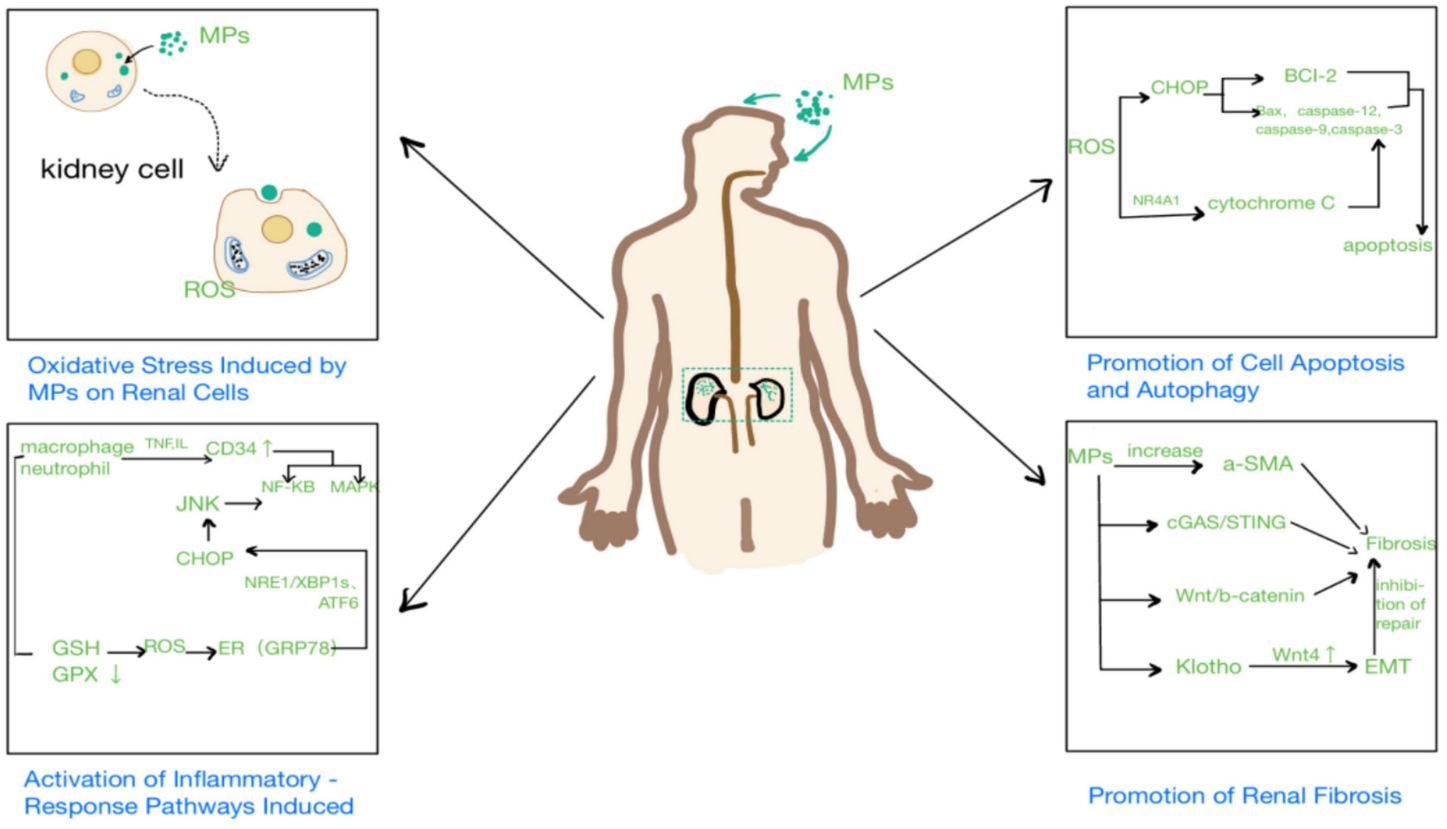
Mechanisms of the toxic effects of microplastics on the kidneys.

Previous studies have shown that inflammation, oxidative stress, and apoptosis are drivers of fibrosis in the kidneys. After exposure to MPs, the expression level of *α*-SMA in the kidneys increases significantly, leading to collagen fiber deposition and accelerating the progression of fibrosis. It was also found that MPs of varying diameters can cause different renal pathophysiological conditions by promoting oxidative stress, inflammation, and fibrosis through circadian rhythm disruption (33). Shen et al. demonstrated that MP exposure can induce DNA damage in the nucleus and mitochondria, resulting in the translocation of dsDNA fragments into the cytoplasm. This process triggers the DNA-sensing adaptor protein STING, activates the cGAS/STING pathway, and then activates NF-κB, which translocates into the nucleus to upregulate the expression of pro-inflammatory cytokines, ultimately promoting fibrosis (49). A study has shown that PS-MPs can cause fibrosis in rats by activating the Wnt/*β*-catenin signaling pathway (50). Recently, Pan et al. found that the abnormal expression of Klotho, induced by MPs, plays a crucial role in mediating kidney fibrosis and tubular senescence. Continuous upregulation of Wnt4 by MPs can induce EMT in epithelial cells of senescent tubules, inhibiting the proliferation and repair of normal epithelial cells (51). This indicates that in the aging kidney model, tubular cells are more susceptible to senescence (Figure 3).

Microplastics have a significant impact on the kidneys. Structurally, they can damage both the glomeruli and renal tubules, leading to podocyte injury and degeneration, and necrosis of renal tubular epithelial cells. The mechanisms underlying these effects are multifactorial and include oxidative stress, inflammation, apoptosis, autophagy, fibrosis, and the generation of extracellular vesicles. Oxidative stress can activate inflammatory signaling pathways, with the resulting increase in inflammatory factor expression further aggravating kidney damage. Apoptosis and autophagy interact to jointly regulate cell fate. Fibrosis alters the structure of kidney tissue, ultimately affecting kidney function.

Although progress has been made in understanding the impact of MPs on the kidneys, several important questions remain.

There is a significant difference in exposure between *in vivo* and *in vitro* experiments. Under the same exposure dose, cells remain active in the in vitro experiments, whereas in vivo experiments show kidney glomerular barrier damage and proteinuria. This indicates that the toxicity of microplastics (MPs) may not only affect kidney cells directly, but also induce organ-level damage and pathological changes, such as the activation of reactive oxygen species (ROS). This can trigger systemic release of inflammatory cytokines like TNF-*α* and IL-6, which circulate and indirectly damage the kidneys. The *in vivo* microenvironment, including inflammation and cascade amplification effects, may potentiate their toxicity. Thus, in vivo studies may be closer to the real physiological environment, but they might be restricted by experimental conditions and ethics.

Compared with microplastics, nanoplastics are more likely to penetrate cell membranes and biological barriers, entering the cell nucleus and mitochondria, and directly damage subcellular structures. Some NPs can be reabsorbed by the renal tubules and accumulate over a long period of time. Due to their large specific surface area, nanoparticles are also more likely to adsorb heavy metals and organic pollutants, potentially leading to higher combined toxicity. Consequently, extensive experimental designs are needed to simulate the toxic effects of NPs.

Current research mainly focuses on the changes in kidney cells after MP exposure. However, research on the mode of entry of MPs, the potential effects of long-term low-dose exposure, and the combined toxic effects with other environmental pollutants is limited. Future studies should use advanced detection techniques to track MP entry into the kidneys, as well as clarify their distribution and targets at cellular and subcellular levels. Long-term, low-dose exposure experiments are needed to simulate real-world environmental conditions and evaluate their chronic toxic effects (including the relationship between exposure duration, dosage, and effect). In addition, more research on the combined effects of MPs and other pollutants is needed to more comprehensively assess their potential threats to kidney health and provide a scientific basis for formulating effective prevention and control measures.
